# Chemistry Combining Elemental Profile, Stable Isotopic Ratios, and Chemometrics for Fine Classification of a Chinese Herb Licorice (*Glycyrrhiza uralensis* Fisch.) from 37 Producing Area

**DOI:** 10.1155/2022/8906305

**Published:** 2022-08-18

**Authors:** Zhongying Lu, Chengying Hai, Simin Yan, Lu Xu, Daowang Lu, Yixin Sou, Hengye Chen, Xiaolong Yang, Haiyan Fu, Jian Yang

**Affiliations:** ^1^Department of Food Engineering, Guizhou Vocational College of Foodstuff Engineering, Guiyang 551400, China; ^2^The Modernization Engineering Technology Research Center of Ethnic Minority Medicine of Hubei Province, College of Pharmacy, South-Central Minzu University, Wuhan 430074, China; ^3^Shanghai Institute of Quality Inspection and Technical Research, Shanghai 201114, China; ^4^College of Material and Chemical Engineering, Tongren University, Tongren 554300, Guizhou, China; ^5^State Key Laboratory Breeding Base of Dao-di Herbs, National Resource Center for Chinese Materia Medica, China Academy of Chinese Medical Sciences, Beijng 100700, China

## Abstract

A method based on elemental fingerprint, stable isotopic analysis and combined with chemometrics was proposed to trace the geographical origins of Licorice (*Glycyrrhiza uralensis* Fisch) from 37 producing areas. For elemental fingerprint, the levels of 15 elements, including Ca, Cu, Mg, Pb, Zn, Sr, Mn, Se, Cd, Fe, Na, Al, Cr, Co, and K, were analyzed by inductively coupled plasma atomic emission spectrometry (ICP-AES). Three stable isotopes, including *δ*^13^C, *δ*^15^N, and *δ*^18^O, were measured using an isotope-ratio mass spectrometer (IRMS). For fine classification, three multiclass strategies, including the traditional one-versus-rest (OVR) and one-versus-one (OVO) strategies and a new ensemble strategy (ES), were combined with two binary classifiers, partial least squares discriminant analysis (PLSDA) and least squares support vector machines (LS-SVM). As a result, ES-PLSDA and ES-LS-SVM achieved 0.929 and 0.921 classification accuracy of GUF samples from the 37 origins. The results show that element fingerprint and stable isotope combined with chemometrics is an effective method for GUF traceability and provides a new idea for the geographical traceability of Chinese herbal medicine.

## 1. Introduction

Licorice, as a widely used herb in traditional Chinese medicine Gancao, is the dried root and rhizome of *Glycyrrhiza uralensis* Fisch. (GUF), *Glycyrrhiza inflata* Batalin (GIB), and *Glycyrrhiza glabra L*. (GGL). It is known as “the king of traditional Chinese medicine (TCM)” and is one of the most commonly used herbs in TCM formulas, which has been registered as one of the four major medicinal plants by Chinese medical administration department [1–3]. Licorice contains flavonoids, saponins, alkaloids, coumarins, and other compounds [4–7], with various pharmacological effects such as antioxidant, anti-inflammatory, immunomodulatory, antiulcer, and antiviral [8–10]. Licorice is widely distributed in the areas of latitude 37°∼47°N and longitude 73°∼125°E, mainly distributed in the arid and semidry early areas of about 40°N latitude [11]. The major medicinal plant of licorice in China is GUF, which is widely distributed in the northwest and northeast provinces [12]. The quality of licorice is believed to be largely influenced by its geographical origin due to the differences in climate, soil, and other ecological environmental factors in different producing areas [13–15]. However, we all know that the chemical composition of licorice depends largely on internal genetic and metabolic factors. There may be great differences even among products of the same origin, so it is necessary to develop some methods to accurately identify the geographical origins of licorice.

In recent years, many methods have been used to distinguish the geographical origin of licorice and other herbs [16, 17], including high performance liquid chromatography [18], gas chromatography [19], nuclear magnetic resonance [20], electron nasal tongue [21], and near infrared spectroscopy [22], which come with high accuracy, but these methods are mainly analyzed by chemical composition. In recent years, stable isotopes have been widely used for origin tracing because of their excellent stability as well as the advantages of fast analysis, high precision, and significant effect. By using the principle of natural fractionation effect of isotopes, samples of different origins show significant differences in the abundance of isotopes of elements (^13^C/^12^C, ^15^N/^14^N, ^18^O/^16^O) in natural substances due to differences in environment, climate, and soil. This information carries the environmental factors, reflects the environmental conditions of the organism and becomes a kind of “natural fingerprint”, which can be used to distinguish substances of different geographical origins [23–25]. There have been many reports on the application of this method to the identification of licorice origins [26–28], but they are limited to the classification of a small number of origins by simple pattern recognition, while licorice origins are widely distributed, so it is necessary to identify licorice by large class number classification.

The so-called fine classification or large-class-number-classification (LCNC) [29] is more complex than the general multiclass classification (usually no more than 10 classes are involved [30] because: (1) a large class number will greatly increase the possibility of class overlap and reduce the classification accuracy of the model; (2) the complexity of data structure and model will increase drastically with the number of classes, which will increase the risk of over-fitting and reduce the generalization performance of the model; and (3) error accumulation becomes an outstanding problem, as the decision-making of multiclass model is usually based on the results of a set of binary classifiers. However, there is a little research devoted to the methods of LCNC [31]. Therefore, it is necessary to adopt a new chemometrics strategy for the fine classification of GUF from a number of geographical origins. The main aim of this work was to investigate the feasibility and performance of using elemental fingerprints and stable isotopic ratios for the classification of GUF geographical origins. Fine classification of GUF from 37 producing areas was performed by comparing the traditional one-versus-rest (OVR) and one-versus-one (OVO) strategies with a new ensemble strategy (ES) to obtain an accurate and effective classification system for quality inspection and control of GUF.

## 2. Materials and Methods

### 2.1. Reagents and Standard Solutions

Standard materials of Al, Cr, Mg, Pb, Zn, Ca, Cu, Mn, Se, Cd, Fe, Na, Sr, Co, K, and Ni were purchased from the National Standard Material Center of China. Isotope reference materials for *δ*^13^C, *δ*^15^N, and *δ*^18^O, including L-glutamic Acid USGS40 (*δ*^15^N = −4.50‰ relative to atmospheric N^2^ and *δ*^13^C = −26.389‰ relative to Vienna Pee Dee Belemnite standard), caffeine USGS61(*δ*^15^N = −2.87‰ relative to atmospheric N^2^ and *δ*^13^C = −35.05‰ relative to Vienna Pee Dee Belemnite standard), Vienna Standard Mean Ocean Water (VSMOW) (*δ*^18^O = 0), and Benzoic Acid IAEA-601 (*δ*^18^O = 23.3‰ relative to VSMOW1 standard), were purchased from the International Atomic Energy Agency (Vienna, Austria). Secondary distilled water was used in preparing standard and sample solutions. Nitric acid (HNO_3_, 65%, w/w %) and hydrogen peroxide (H_2_O_2_, 30%, w/w %) for sample digestion were purchased from Sinopharm Chemical Reagent Co. Ltd. (Shanghai, China).

### 2.2. Collection of GUF Samples

**Figure 1:**
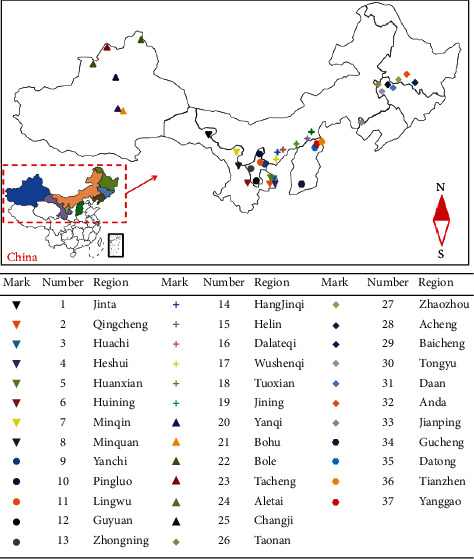
The geographical origins of 37 classes of GUF.

Dried GUF samples were purchased from the local herbalists of 37 producing areas in China. All the GUF samples were cultivated artificially and harvested with a growth period of 2 and 3 years. The geographical locations of the 37 producing areas are plotted in Figure 1. From each producing area, 30 objects were collected, making a total of 1110 objects. Classes 1–8, 9–13, 14–19, 20–25, 26–33, and 34–37 belong to Gansu, Ningxia, Neimenggu, Xinjiang, the three Northeast provinces, and Shanxi province, respectively.

### 2.3. Preparation of Sample Powder

Before elemental and stable isotope ratio analysis, the GUF rhizome was cleaned and washed using tap water followed by deionized water. The cleaned rhizome was cut into thin slices, put into an electro-thermal blast drying oven, and dried for 24 hours at a temperature of 50°C to a constant weight. Subsequently, the fully dried slices were crushed into powder with a grinder and filtered through a 100-mesh sieve.

### 2.4. Digestion and Elemental Analysis

For sample digestion, about 0.5 g dry GUF powder was put into a Teflon digestion tank with 9 mL nitric acid and 3 mL H_2_O_2_ for 24 hours. The solution was heated to 60°C and kept for 5 minutes and then to 160°C for 10 minutes until no more white smoke rose. The colourless and transparent solution was cooled naturally, and after 2 hours of standing it was transferred to a 50 mL volumetric flask to prepare the test solutions.

Levels of the 15 inorganic elements in GUF were analyzed using a Shimadzu ICPS-7510 sequential plasma emission spectrometer (Shimadzu, Kyoto, Japan). The working parameters of the spectrometer were as follows: power: 1300 W; plasma flow rate: 15 L min^−1^; carrier gas flow rate: 0.8 L min^−1^; auxiliary flow rate: 0.2 L min^−1^; atomization flow rate: 0.8 L min^−1^; pump flow rate: 1.5 mL min^−1^; axial observation distance: 15 mm; and the instrumentation stabilization time of 30 s. Simultaneously considering the intensity, interference, and signal stability, the selected analytical line for each element was listed in Table 1. The elemental levels were determined by standard curves.

### 2.5. Stable Isotopes Ratios Analysis

Stable isotope ratios were analyzed on an elemental analyzer-isotope-ratio mass spectrometer (EA-IRMS) (Thermo Fisher Scientific, Waltham, MA, USA). For *δ*^13^C and *δ*^15^N analysis, about 0.3 mg of the dry powder was weighed, wrapped in a tin cup, and transferred to the fast combustion furnace through the automatic sample feeder of the element analyzer. The resultant CO_2_ and N_2_ were dried, separated, distilled, and analyzed by IRMS. The analytical conditions were: oxidation furnace temperature: 960°C; flow rate of carrier gas: 100 mL min^−1^; sample purging gas flow rate: 225 mL min^−1^; oxygen flow speed: 175 mL min^−1^; the time of oxygen injection: 3 s; and the temperature of the gas chromatography column was 50°C. For *δ*^18^O analysis, about 0.3 mg of the dry licorice powder was weighed, wrapped in a silver cup, and transferred to the high-temperature cracking furnace. The resultant CO was dried, separated, distilled, and analyzed by IRMS. The analytical conditions were: cracking furnace temperature: 1400°C; flow rate of carrier gas: 100 mL min^−1^; sample purging gas flow rate: 150 mL min^−1^; and the temperature of the gas chromatography column was 90°C.

### 2.6. Chemometrics Analysis

Outliers are abnormal objects that deviate from the bulk of the other observations. For classification models, outliers in the training set will cause bias in model estimation, while outliers in the test set will lead to misleading results when evaluating the model's performance. Therefore, outlier diagnosis is required to detect and exclude unusual objects before model building. Robust statistical methods with dimension reduction techniques are required to detect outliers. The robust Stahel-Donoho estimate (SDE) of outlyingness [32] was computed for outlier diagnosis. The SDE outlyingness is based on a large number of random projections of the high-dimensional objects and robust estimates of location as well as scale by the median and median absolute deviation (MAD), respectively.

The DUPLEX algorithm [33] was used to divide the measured data into a training set and a test set. The aim of this algorithm was to obtain a representative training set in such a way that the objects are scattered uniformly in the experimental space. Because the distribution of GUF samples from each producing area was not the same, the DUPLEX method was performed separately for each class. The training and test samples of each class were combined to form the final training and test sets, respectively.

Usually, multiclass classification can be performed by combining a multiclass strategy with a two-class classifier. In this work, the traditional one-versus-rest (OVR) [34] and one-versus-one (OVO) [35] strategies and a new ensemble strategy (ES) were used as multiclass strategies. For two-class classifiers, the linear partial least-squares discriminant analysis (PLSDA) [36] and the nonlinear least-squares support vector machine (LS-SVM) [37] were used.

**Figure 2:**
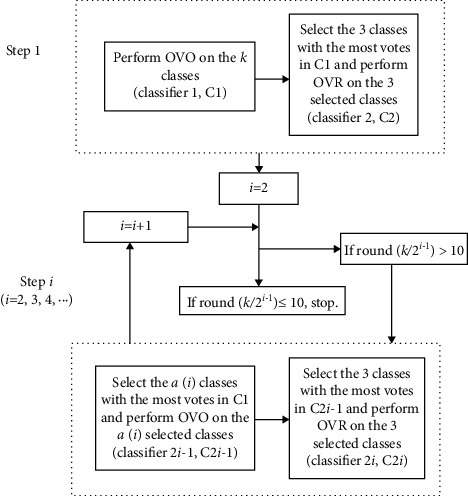
The principle of the ensemble strategy (ES) for fine classification.

The multiclass strategy forms the framework of two-class classifiers and combines the results of the latter for the final decision-making. Suppose there are k classes to be classified. The OVR strategy develops k binary one-versus-(k-1) models, where each class is discriminated from the rest k-1 classes. For the i th (*i* = 1, 2, 3,…, and k) model, class i is labeled as +1 and the other (k-1) classes are labeled as -1. A new object is predicted sequentially by the above k one-versus-(k-1) classifiers and assigned to the class that has the highest predicted response value. For OVO, binary classifiers are developed between each pair of the k classes, so there are k (k-1)/2 binary classifiers in all. For a new object, it is predicted by the k (k-1)/2 classifiers sequentially, and each classifier assigns the object to one of the two classes, and finally, the decision-making is done by a max-wins voting strategy. As a combination of OVR and OVO, the principle of the new ES strategy is shown in Figure 2. In step 1, OVO is performed for all k classes (classifier 1, C1) and OVR is performed for the 3 classes with the most votes in C1 (classifier 2, C2). At step i (*i* = 2, 3, 4, ......), the class (*α*(*i*)) with the most votes in decreasing order in C1 is selected and two submodels are developed: (1) a new OVO classification model (classifier 2i-1, C2i-1) is developed on the chosen class *α*(*i*); (2) a new OVR classification model (classifier 2i, C2i) is developed on the 3 classes with the most votes in C2i-1. The procedure is performed at *α*(*i* + 1) *b* 10 (*i* = 2, 3, 4, ...) when it stops and develops 2i subclassifiers to classify the new object. In this paper, *α*(*i*) is defined as follows:

(1) $$\begin{array}{c} \alpha \left(i\right)=\text{round}\left(\frac{k}{2i}-1\right),\;i=2,3,4\ldots , \end{array}$$

where the operator “round” means rounding.

Finally, for a new object, the predictions of the 2i subclassifiers will be combined to make the final decision via the max-wins voting strategy. In most cases, the new object will be uniquely assigned to the class that receives the most votes. However, when more than one class receives the most votes, an additional OVR classification model will be developed to uniquely assign the object to one of these classes.

Regarding the submodels of ES, for all OVO submodels except C1, the *α* (*i*) class with the most votes is selected from C1, where *α* (*i*) is k/2, k/4, k/8, and so on. The trade-offs are as in Equation (1). When a new object is predicted by OVO, the more votes a class receives, the more likely it is to come from that class. Thus, with the minimum value of *α* (*i*) set to 10, subsequent OVO submodels can include the true class labels with a very high probability. In this way, even if a very large number of classes are to be classified, ES can still have a modest total number of submodels. The serial OVR submodel is developed on 3 selected classes because the performance of OVR decreases dramatically when the number of classes increases.

### 2.7. Software

All the data analysis and chemometric modeling were performed in MATLAB 7.0.1 (Mathworks, Sherborn, MA, USA). The DUPLEX algorithm was performed using the TOMCAT toolbox [38]. The LS-SVM algorithm was developed using the toolbox LS-SVMlab v1.8. All the other data analysis algorithms were performed based on in-house computational coded scripts written by the authors in MATLAB.

## 3. Results and Discussion

### 3.1. Data of Elemental Profiles and Stable Isotopic Ratios

The analysis data of the 15 elements as well as the 3 stable isotopic ratios for GUF samples were summarized in Table 2. Two elements, Se and Cd, were not detected in the current analytical conditions. For the other 13 elements, except Mg, Cu, and Mn, each of the other 10 elements has a wide range of content, indicating the soils of different producing areas have very different elemental profiles. For *δ*^13^C, previous study [39] indicated that its value tends to increase with a higher altitude. Changes in altitude cause changes in other environmental factors, such as precipitation, light, temperature, and atmospheric pressure, which can influence the morphology, physical properties, and photosynthetic gas exchange, and ultimately the *δ*^13^C value of plants. With similar ambient humidity, the difference in *δ*^13^C samples could be mainly attributed to variations in altitudes. For *δ*^15^N, previous studies [40] found that the nitrogen in plants depends on the nitrogen pool in the soil (nitrate and ammonia), whose nitrogen isotopic compositions depend on its geographical and climatic conditions and are related to agricultural fertilization. The level of *δ*^18^O was found to decrease with latitude due to fractionation.

**Figure 3:**
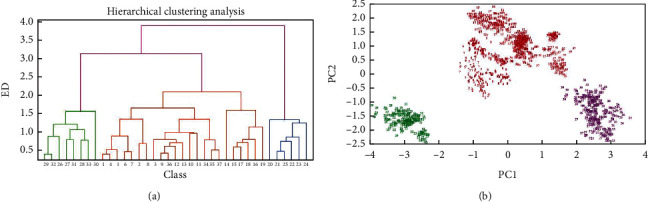
(a) HCA of 37 classes of GUF samples (average) and (b) the first two PCs for all the GUF samples.

For all the data analysis, each feature was auto-scaled to have zero center and the unit length to reduce the influence of data scales. To demonstrate the data distribution, hierarchical clustering analysis (HCA) using the Euclidean distance (ED) and principal component analysis (PCA) were performed on the auto-scaled data (Figure 3). Both HCA and PCA clearly indicate that the total data could be clustered into three big groups, that is, Xinjiang province (classes 20–25), Central-north (classes 1–8, 9–13, 14–19, and 34–37), and Northeast (Classes 26–33). HCA could discriminate against big groups, but some classes from the same provinces were not tightly linked; e.g., classes 1–8 come from Gansu province, while class 3 was linked to classes 9–13 from Ningxia province. It cannot be assumed that the confusion of classes across provinces by HCA is wrong, because the soil variations within a province may be larger than those between two provinces. However, the HCA results indicate that using the ED of raw features may be insufficient for accurate classification of a large number of classes. The first two principal components (PCs) explained 81.06% of the total data variances, and projection of the 16 features onto the first 2 PCs can provide some separation of the two classes, but the separation is insufficient and overlapping still exists. Chemometrics models were still demanded to achieve a more accurate classification of the 37 classes.

### 3.2. Chemometrics Classification Results

Outlier diagnosis and data splitting were shown in Table 3. For outlier diagnosis, robust SDE analysis was performed on each of the 37 classes. In this work, the number of random projections of SDE was 500. According to the 3*σ* rule, an SDE outliervalue above 3 can be seen as an indicator of an outlier. As a result, each of classes 20, 27, and 37 had one outlier detected and were excluded from further data analysis. Therefore, each of classes 20, 27, and 37 had 29 objects left and all the other classes had 30 objects. After the removal of outliers, the DUPLEX algorithm was performed on each class to divide it into 20 training and 10 test objects (9 for classes 20, 27, and 37), which were combined to generate the final training and prediction sets. Finally, a training set of 740 objects and a test set of 367 objects were obtained for the training and validation of classification models.

With the 3 multiclass strategies and 2 binary classifiers, 6 multiclass classification systems were developed and compared, including OVR-PLSDA, OVO-PLSDA, ES-LS-SVM, OVO- LS-SVM, OVR- LS-SVM, and ES-LS-SVM. Monte Carlo Cross Validation (MCCV) [41] splits the data repeatedly and randomly into calibration and test sets, avoiding unexpected performance results. The complexity of the models is optimized by performing internal k-fold cross-validation on each available calibration set. To take into account the predictive performance when applying the models to new samples, each model is validated with a separate test set. For PLSDA, MCCV was used to determine the number of latent variables. The number of random data splitting in MCCV was 100 and for each splitting, 80% of the training data were used for training a PLSDA model and the other 20% were used for validation. Thelargest number of LVs to be screened was set to be 10, and the model with the lowest misclassification rate of MCCV (MRMCCV) was selected using the following equation:

(2) $$\begin{array}{c} \mathrm{MRMCCV}=\frac{\sum_{i=1}^{100}m_{i}}{\sum_{i=1}^{100}v_{i}}. \end{array}$$

Where *v*_*i*_ is the number of validation objects and *m*_*i*_ is the number of misclassified objects.

For LS-SVM, two parameters, the kernel width parameter *σ* and the regularization parameter *γ* need to be optimized. *σ* adjusts the data confidence and the nonlinear nature of the model. A smaller *σ* corresponds a narrower kernel, which can obtain a model with a more complex nonlinear solution. *γ* balances the tradeoff between training accuracy and structural risk. In this work, *σ* and *γ* were optimized using a grid search by 10-fold cross validation in the toolbox LS-SVMlab v1.8. The combination of (*σ*, *γ*) in LS-SVM was optimized to achieve the lowest root mean square error of cross validation (RMSECV).

The classification results by different multiclass classification systems are listed in Table 3. With OVR-PLSDA, the total classification accuracy of the 37 GUF was 0.776 with the raw data. The poor prediction performance of OVR-PLSDA can be attributed to the following aspects: by examining the binary PLSDA models, most of the 1-VS-36 PLSDA classifiers included 10 LVs (the maximum LVs number screened). A high model complexity generally leads to a bad generalization performance. Because the sizes of two groups are severely unequal (1-VS-36), the estimation of binary PLSDA boundaries tends to have a bias, which could cause extra uncertainty, although it has been corrected by the weight centering strategy. Moreover, because OVR selects the largest response among 37 1-VS-36 classifiers, the final results will be severely affected by class overlapping and the accumulation of model errors. The OVR-LS-SVM obtained slightly better classification results with a total classification accuracy of 0.784, which can be attributed to the extra model flexibility of LS-SVM compared with linear PLSDA.

For the OVO-PLSDA, the total classification accuracy was 0.866 and the average LVs number of OVO-PLSDA submodels was 3.15. Compared with OVR-PLSDA, the performance of OVO-PLSDA was much less influenced by the increasing class number. This can be attributed to the model simplicity of OVO subclassifiers. Moreover, for the training data set, because the class sizes of the 37 classes are equal (20 objects for each class), the estimation of the classification boundaries of subclassifiers by OVO-PLSDA would be more reliable than by OVR-PLSDA. The OVR-LS-SVM obtained a comparable classification performance with a total classification accuracy of 0.880, indicating the multiclass strategy had more important influence on the final results than the selection of binary models for PLSDA or LS-SVM. In addition, cross-validation was used to verify whether the models were over-fitted, and the results showed that the cross-validation of the six models were 78.6%, 86.0%, 92.3%, 77.8%, 87.4%, and 92.0%. ES-PLSDA and ES-LS-SVM have high cross-validation accuracy, indicating that these two models have better classification performance in the classification of large class numbers of licorice.

As shown in Figure 2, for a 37-class problem, the ES strategy would reduce the number of classes in 3 steps (from 37 classes to 19 classes and finally to 10 classes) and needs 6 models. The total classification accuracy of 37 GUF was 0.929 and 0.921 for ES-PLSDA and ES-LS-SVM, respectively. In the first step, the object was wrongly assigned to class 35 by OVO-LS-SVM and to class 34 by OVR-LS-SVM, respectively. In the second step, by reducing the number of classes from 37 to 19, the object was wrongly assigned to class 10 by OVO-LS-SVM but correctly classified by OVR-LS-SVM. In the third step, by reducing the number of classes from 19 to 10, both OVO-LS-SVM and OVR-LS-SVM could correctly classify the test object. The results indicate that ES could enhance the prediction accuracy by combining OVO and OVR and sequentially reducing the number of classes.

**Figure 4:**
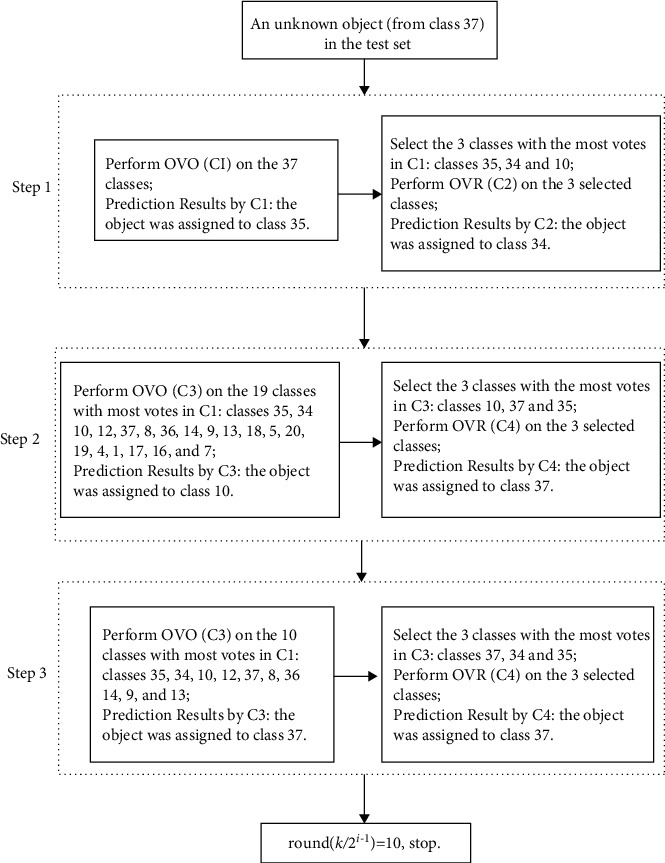
The flowchart of ES-LS-SVM to predict a test object (from class 37).

To demonstrate how ES achieved the improvement in classification accuracy over OVR and OVO, the classification flowchart of a test object (from class 37) by ES-LS-SVM is demonstrated in Figure 4. In classifier 1 (C1), 37 classes were performed using OVO-LS-SVM. The result showed that the object was wrongly assigned to class 35. For classifier 2 (C2), the 3 selected classes had not included the actual class label (class 37) to develop the 3-class-OVR models, and the result showed that they had wrongly classified it to class 34. For classifier 3 (C3), using the 19 classes with the highest votes in C1 to perform OVO-LS-SVM and wrongly classified it as class 34. For classifier 4 (C4), the 3 selected classes included the actual class label (class 37) to develop the 3-class-OVR models and obtain the correct predictions. Especially, using the first 10 classes with the most votes rather than all the 37 classes, the reduced OVO model correctly recognized the class label. The results indicate that ES could combine the advantages of both OVR and OVO and the sequential reduction of class number as shown in Figure 2 is effective to enhance the prediction accuracy of both submodels and the final model.

## 4. Conclusions

The feasibility of combining elemental fingerprints, stable isotopic ratios, and chemometrics for fine classification of GUF geographical origins was studied. Fifteen mineral elements (Al, Cr, Mg, Pb, Zn, Ca, Cu, Mn, Se, Cd, Fe, Na, Sr, Co, and K) were analyzed by ICP-AES and 3 stable isotopes, including *δ*^13^C, *δ*^15^N, and *δ*^18^O, were analyzed by isotope mass spectrometry. Three multiclass strategies, including OVO, OVR, and ES, were combined with two binary classifiers, PLSDA and LS-SVM, to develop LCNC systems. Compared with OVO and OVR, the ES method could improve the classification capacity and obtain better classification results. Especially, the accuracy of ES-PLSDA and ES-LS-SVM was 0.929 and 0.921, respectively. Chemometrics analysis of elemental levels and stable isotopes can be used as an effective method for the tracing of herbal origins.

## Figures and Tables

**Table 1:** Selected analytical wavelengths for the 15 different elements.

Elements	Wavelength (nm)	Elements	Wavelength (nm)
Zn	213.8	Sr	407.8
Ca	393.4	Co	236.4
Cu	324.8	K	766.5
Mn	257.6	Se	196.0
Al	396.2	Cd	228.8
Cr	267.7	Fe	238.2
Mg	280.2	Na	589.6
Pb	220.4	—^a^	—

‘a' represents nondetected.

**Table 2:** Summary of elemental analysis and stable isotopic ratios for GUF samples from 37 different geographical origins.

Items^a^	Lowest of average^b^	Highest of average	Sd of average
Se	–^c^	–	–
Cd	–	–	–
Fe	45	809	211
Na	319	4615	1123
Sr	0.912	8.228	1.9
Co	0.587	4.729	1.1
K	3050	11330	2091
Al	33	415	116
Cr	0.315	20.8	5.5
Mg	1639	4578	819
Pb	0.039	0.408	0.09
Zn	2.965	22.80	5.1
Ca	857	6623	1270
Cu	3.25	10.77	2.1
Mn	8.56	30.75	6.7
*δ*13C	–34.1	–27.1	1.8
*δ*15N	–4.97	–2.15	0.8
*δ*18O	10.1	15.3	1.4

‘a' represents the units of elemental levels and stable isotopic ratios, which are *μ*g/g dry weight and %, respectively. ‘b' represents the average of the 30 objects from each geographical origin. ‘c' represents nondetected.

**Table 3:** Classification of 37 GUF geographical origins by different multiclass classification systems.

Models	Training set errors rate (%)	Prediction set accuracy (%)	Cross validation accuracy (%)
OVR-PLSDA	79.0	77.6	78.6
OVO-PLSDA	87.7	86.6	86.0
ES-PLSDA	91.5	92.9	92.3
OVR-LS-SVM	62.5	78.4	77.8
OVO-LS-SVM	70.9	88.0	87.4
ES-LS-SVM	86.3	92.1	92.0

‘a' represents MRMCCV for PLSDA and RMSECV for LS-SVM.

## Data Availability

The data used to support this study are available from the corresponding author upon request.

## Abbreviations

ICP-AES: Inductively coupled plasma atomic emission spectrometry
IRMS: Isotope-ratio mass spectrometer
OVR: One-versus-rest
OVO: One-versus-one
ES: Ensemble strategy
PLSDA: Partial least squares discriminant analysis
LS-SVM: Least squares support vector machines
GUF: *Glycyrrhiza* uralensis Fisch
GIB: *Glycyrrhiza* inflata Batalin
GGL: *Glycyrrhiza* glabra *L*
TCM: Traditional Chinese medicine
LCNC: Large-class-number-classification
SDE: Stahel-Donoho Estimate
MAD: Median absolute deviation
HCA: Hierarchical clustering analysis
ED: Euclidean distance
PCA: Principal component analysis
MCCV: Monte Carlo cross validation
MRMCCV: Misclassification rate of MCCV
LVs: Latent variables
RMSECV: Root mean square error of cross validation.
